# Promising Gene Delivery Properties of Polycations Based on 2-(N,*N*-dimethylamino)ethyl Methacrylate and Polyethylene Glycol Monomethyl Ether Methacrylate Copolymers

**DOI:** 10.3390/polym15143036

**Published:** 2023-07-13

**Authors:** Tatiana P. Loginova, Irina A. Khotina, Yurii A. Kabachii, Sergei Yu. Kochev, Vyacheslav M. Abramov, Valentin S. Khlebnikov, Natalia L. Kulikova, Yaroslav O. Mezhuev

**Affiliations:** 1 A.N. Nesmeyanov Instituite of Organoelement Compounds of Russian Academy of Sciences, Vavilova Street 28, 119334 Moscow, Russia; khotina@ineos.ac.ru (I.A.K.); kabachi@ineos.ac.ru (Y.A.K.); kochew@ineos.ac.ru (S.Y.K.); 2 JSC Institute Immunological Engineering, Nauchnaya street 1, 142380 Lybuchany, Moscow District, Moscow Region, Russia; slavab@mail.ru (V.M.A.); vkhleb@mail.ru (V.S.K.); nlk.05@mail.ru (N.L.K.); 3 Department of Biomaterials, Mendeleev University of Chemical Technology of Russia, 125047 Moscow, Russia

**Keywords:** polycations, DNA, transfection, HEK 293, interpolymer complex, DNA carriers, gene delivery

## Abstract

Cationic copolymers based on 2-(N,*N*-dimethylamino)ethyl methacrylate and polyethylene glycol monomethyl ether (pDMAEMA-co-PEO) with different molecular weights have been synthesized. Their physicochemical properties were studied by NMR spectroscopy, sedimentation, and potentiometric titration. According to the data of potentiometric titration for the synthesized pegylated cationic copolymers, the apparent dissociation constants were determined in the pH range from 4.5 to 8.5. The physicochemical properties of interpolyelectrolyte complexes of these polycations with circular DNA (IPEC DNA) were also studied by dynamic light scattering, electrophoretic mobility, and TEM methods. It has been established that the diameter and electrokinetic potential (ζ-potential) of interpolyelectrolyte complexes can be varied over a wide range (from 200 nm to 1.5 μm and from −25 mV to +30 mV) by changing the ratio of oppositely charged ionizable groups in pegylated cationic copolymers and DNA, as well as by regulating medium pH. The resistance of the IPEC DNA/polycation complex to the action of nucleases was studied by electrophoresis in agarose gel; the cytotoxic effect of the polymers in vitro, and the efficiency of penetration (transfection) of IPEC DNA with PDMAEMA-co-PEO-polycations into eukaryotic cells of a cell line derived from human embryonic kidneys HEK 293 in vitro.

## 1. Introduction

In recent decades, research aimed at developing new effective means of targeted delivery of biologically active substances, including vaccine genes and DNA, to target cells and organs is being carried out [1,2,3,4,5,6]. DNA vaccine technologies are extremely relevant for the design of safe and effective vaccines against the emergence of infectious diseases and pathogens also they present a promising alternative vaccine platform that has the potential to treat many diseases, such as cancers, atherosclerosis, and diabetes [7,8,9]. The necessary plasmid constructs could be quickly produced without any protein purification, as the antigens encoded by DNA vaccines are expressed in vivo [5,10]. 

This enables a quick screening and optimization of many alternative antigens based on their immunogenicity and data on protection obtained in animal studies. DNA inoculation could induce both humoral and cellular immune responses, besides it is important to note that for non-invasive DNA vaccine application, mucosal surfaces represent the easiest and most logistical pathway. This enables the use of a wide spectrum of delivery systems like liposomes, microparticles, or live bacterial vectors [11].

DNA immunization with vaccines is usually performed intramuscularly using gene-gan technology. However, even in this case, multiple injections of the material (5–6 or more injections) are required to achieve the expected effect. This is probably due to the loss of DNA material under the action of nucleases, cell injury, and low potential for targeted delivery of material to antigen-presenting cells [12,13]. 

The design of DNA vaccines against bacterial pathogens is less fully understood, perhaps because protective immunity to most bacterial infections is thought to be highly dependent on the humoral immune response. But, despite the obvious success in this direction, a number of principal problems have not yet been solved [14,15]. The difficulty of delivering DNA vaccines to target immune cells is a major challenge that prevents advances in DNA vaccines and limits their clinical use because that obstructs the stimulation of strong antigen-specific immune responses in humans [16,17]. The effectiveness of DNA vaccines largely depends on successful transfection, which must avoid the loss of DNA material under the action of nucleases, and overcome inactivation through non-specific interactions with other proteins and a lot of extracellular and intracellular barriers [18]. 

One of the ways to improve the efficiency of DNA vaccine technologies is the development and use of delivery systems based on polymer nanoparticles [19,20,21]. Enhancement of the immunogenicity of the DNA payload, minimizing toxicity, providing targeted delivery to antigen-presenting cells, the introduction of DNA uptake and nuclear entry, and improving the overall antigen-specific immune response are offered by polymer NPs (nanoparticles) delivery systems. Having ranges of about 10–500 nm, polymer NPs enable them to be readily taken up by cells, as well as to avoid RES (reticuloendothelial system) clearance [22,23,24]. Of particular interest for the immobilization of drugs and DNA are polyelectrolyte complexes, which are more resistant to negative influences, compact, and able to penetrate into cells compared to free DNA [25,26,27,28,29,30]. Due to the pH sensitivity of polyelectrolytes, the rate of DNA release in target cells can be controlled [31]. In addition, it is known that non-ionic polymers often have a low affinity for biological membranes. 

Among the currently known cationic polymers used for gene delivery, there are a small number of polymers, such as chitosan, poly(2-(N,*N*-dimethylamino)ethyl methacrylate), polyethyleneimine, which provide effective targeted delivery of DNA plasmids and are, at the same time, substances that affect the cell membrane [32,33,34,35,36,37,38,39,40,41,42,43,44,45]. It should be noted that cationic polyelectrolytes with a high charge density also have high cytotoxicity, which can be reduced by introducing polyethylene glycol (polyethylene oxide—PEO), which has good water solubility and biocompatibility, into their composition. On the other hand, the introduction of hydrophilic PEO fragments into DNA carrier copolymers can promote the development of adverse immune responses for a small number of patients capable of producing PEO—directed immunoglobulins [46]. However, for the vast majority of patients, pegylated carriers are non-immunogenic and are gold standards for the delivery of pharmacologically active substances. 

Although few studies have been devoted to the study of the properties of pegylated cationic polyelectrolytes [47,48,49,50], it is obvious that there is a need to search for new synthetic strategies that expand the range of DNA carrier polymers. Therefore, this article is aimed at the synthesis of new functional cationic copolymers of 2-(N,*N*-dimethylamino)ethyl methacrylate and polyethylene glycol monomethyl ether methacrylate. The developed approach makes it possible to simultaneously control the properties of cationic DNA carrier copolymers, both by varying the ratio of 2-(N,*N*-dimethylamino)ethyl methacrylate and polyethylene glycol monomethyl ether methacrylate residues in macromolecules and by changing the molecular weight of hydrophilic PEO fragments. For the synthesized pegylated cationic copolymers, the pH sensitivity was studied, the hydrodynamic diameters were determined, and the electrophoretic mobility of interpolyelectrolyte complexes with cDNA was determined. The resulting interpolyelectrolyte DNA complexes were visualized by electron microscopy, and the efficiency of DNA delivery to eukaryotic cells of the HEK 293 line using synthesized pegylated cationic macromolecular carriers was also evaluated. 

## 2. Materials and Methods

Methacryloyl chloride (97%, Aldrich, St. Louis, MO, USA), polyethylene glycol monomethyl ether (PEG or PEO M_n_ = 2 kD, Aldrich), triethylamine (TEA, Fluka (Charlotte, NC, USA), >99.5%) were used as received. 2-(N,*N*-dimethylamino)ethyl methacrylate (DMAEM, 98% Aldrich) was degassed by a triple freeze–defreeze cycle and distilled in a vacuum into a Schlenk tube, and stored in a refrigerator freezer under argon. 2,2′-Azobis(isobutyronitrile) (AIBN, pure) was purified by crystallization from methanol and stored in a refrigerator. Methylene chloride (CH_2_Cl_2_) was distilled over CaH_2_.

### 2.1. Synthesis of Polyethylene Glycol Monomethyl ether Methacrylate (PEGMA)

PEGMA was synthesized through a modified method described in [51]. The experiment was carried out in argon. In a three-necked flask equipped with a magnetic stirrer and a dropping funnel, 4.66 g (3.33 mmol) of polyethylene glycol monomethyl ether, 47 mL of CH_2_Cl_2_, and 1.3 mL (9.32 mmol) of TEA (triethylamine) were loaded. The mixture was cooled in a bath to 0 °C. Then a solution of 910 μL (9.32 mmol) methacrylic acid chloride in 7 mL CH_2_Cl_2_ was added dropwise for 15 min. Then the cooling bath was removed, the mixture was allowed to warm up to room temperature, and the mixture was stirred for 2.5 h.CH_2_Cl_2_ was distilled off in the vacuum of a water jet pump to a volume of 15–20 mL. The precipitation that formed was separated by filtration. The filtrate was poured into cold (−15 °C) diethyl ether. The formed precipitate was washed on the filter with ethyl ether, and dried in a vacuum of 10^–3^ mm Hg for 4 h. PEGMA yield was 4.1 g. NMR^1^H (CDCl_3_) δ, ppm: 3.37 (3H, OCH_3_), 3.63 (2H, OCH_2_), 4.29 (2H, CH_2_OCO_2_), 5.56 and 6.12 (2H, =CH_2_).

### 2.2. Synthesis of Poly(2-(N,N-Dimethylamino)ethyl Methacrylate)-co-PEGMA) (PDMAEMA-co-PEO)

In a typical experiment (PDMAEMA-co-PEO-1), 1 mL (0.933 g, 5.93 mmol) of DMAEMA, 2.82 mg (17.1 × 10^−3^ mmol) of AIBN, 175.0 mg (2.39 mmol) of PEGMA and 1.2 mL of ethanol were loaded into a Schlenk tube equipped with a magnetic stirrer. The mixture was degassed by a threefold freeze–defreeze cycle in a vacuum, the tube was filled with argon and stirred for 50 h at 70 °C. The polymer was isolated as a hydrochloride by dissolving in water with the addition of 0.52 mL of 11.5 M HCl and purified from residual monomers and excess HCl by washing with demineralized water in a dialysis bag. The solution of the washed polymer was filtered on a porous filter, water was removed under reduced pressure, and dried in a vacuum at 50 °C for 4 h. At the end of the procedure, the copolymers separated from water were additionally reprecipitated from the CH_2_Cl_2_ solution into hexane. To obtain products of different molecular weights, the amount of MPEG introduced into the reaction was varied. The yields of PDMAEMA-co-PEO were 80–85%. Based on the results of the molecular masses study by the sedimentation method for four copolymers, M_SD_ = 165,000 (PDMAEMA-co-PEO-1), 228,000 (PDMAEMA-co-PEO-2), 392,000 (PDMAEMA-co-PEO-3), and 620,000 (PDMAEMA-co-PEO-4).

### 2.3. Characterization

#### 2.3.1. NMR Spectroscopy

All NMR spectra were recorded on a Bruker spectrometer with an operating frequency of 400.13 MHz for ^1^H nuclei and 100.1 MHz for ^13^C nuclei. CDCl_3_ was taken as a solvent.

#### 2.3.2. Sedimentation

The molecular weights of copolymers having a branched molecular structure were determined by sedimentation analysis on an MOM 3180 analytical ultracentrifuge using Filpot–Swensson differential optics. The experiments were carried out in a two-sector cell, one sector of which was filled with a solvent and the other with a solution. Then, 0.2 *N* NaCl was used as a solvent to suppress possible dissociation. The rotor speed was varied in the range of 8000–50,000 rpm (50,000–200,000 *g*). The temperature of the experiments was 25 ± 0.1 °C. For the studied samples, the sedimentation coefficients Sc, and diffusion coefficients D_C_ for four concentrations in the range of 0.5–1.0% were determined. By extrapolating these values to C → 0, the true values of S_0_ and D_0_ were found, which were used further to calculate the molecular weights M_S_. The specific partial volume *V* and solvent density ρ_0_ required for calculating the molecular masses from sedimentation data were determined pycnometrically.

#### 2.3.3. Potentiometric Titration

Potentiometric titration of 0.1% aqueous solutions of polycations in 0.15 M NaCl was carried out on an Inolab pH Level 1 potentiometer in a thermostated cell at a temperature of 20 °C. An HCl solution (0.1 mol/L) was used as a titrant. The dependence of the dissociation constants of partially ionized polycations on the degree of ionization of the amino groups of polycations (α) was determined by potentiometric titration.

pK_app._ = pH + log(α/1-α), where pK_app._ is the apparent dissociation constant of polycations. Ionization degree α—concentration of ionized amino groups/total concentration of ionogenic groups.

#### 2.3.4. Dynamic Light Scattering

Measurements of hydrodynamic particle diameters were carried out on a Brookhaven 90 Plus instrument, at a fixed angle (90°). Light intensity fluctuations were recorded using the Brookhaven90 Plus correlator (Brook haven Instruments Company, Holtsville, NY, USA). To process the obtained data and calculate the particle diffusion coefficients, the software provided by the device manufacturer was used. The hydrodynamic radii were calculated using the Stokes equation in the approximation of spherical particles

#### 2.3.5. Electrophoretic Mobility

The electrophoretic mobility of DNA complexes with polycations was measured using laser microelectrophoresis on a Brookhaven 90 Plus device (Brookhaven Instruments Company) according to the standard procedure in a thermostated cell. Mathematical data processing was carried out using software provided by the manufacturer. DNA complexes with polycations for measuring the Z-potential were obtained as follows: the DNA solution was diluted with PBS (10 mM Na-phosp, 0.15 M NaCl pH 7.2) to a concentration of 20 μg/mL, the calculated amount of the polymer dissolved in water was added, mixed on a vortex for ~20 sec and incubated at room temperature for 30 min.

#### 2.3.6. Determination of Cytotoxicity of Polycation-DNA Complexes

The cytotoxicity of polycations and DNA/polycation complexes was determined using the MTT method [52] based on the intracellular effect of mitochondrial activity. The calorimetric MTT method is based on the selective ability of living cells to reduce 3-(4,5-dimethyl-2-thiazolyl)-2,5-diphenyl-2H-tetrazolium bromide (MTT) with the formation of a dark purple precipitate. The cell line L929, which is most often recommended for studying the cytotoxicity of polymers, was used in the experiment [53]. Cells were seeded into 96-well plates (Costar) with a density of 3 × 10^4^ per ml in DMEM medium (PanEco, Russia) supplemented with 10% fetal calf serum (HyClon). After 24 h of growth at 37 °C in an atmosphere of 5% CO_2_, the culture medium was replaced with a fresh one with the addition of appropriate dilutions of polycations and DNA/polycation complexes and incubated under similar conditions for 24 h. Then MTT (“Sigma”) was added to the medium at a concentration of 0.5 mg/mL. After 4 h, the unreacted solution was taken and the precipitate that formed was dissolved in 100 μL of isopropanol. The results of the experiment were recorded spectrophotometrically on a photomicrocalorimeter (Wallac Victor2 1420 Multilabal TM Counter, Perkin Elmer, Hongkong, China) at wavelengths of 570 nm and 620 nm. Relative cell survival (%) was calculated as A[experiment]/A[control]·100, where untreated samples served as controls.

#### 2.3.7. Transfection of Polycation PDMAEMA-co-PEO/DNA Complexes into Eukaryotic Cells

On the eve of transfection, a suspension of HEK 293 cells (a cell line derived from human embryonic kidneys, Company “Sputnik”, Moscow, Russia) was inoculated into a 12-well plate (Costar) in DMEM without serum and without antibiotic addition. The inoculum dose was determined so that after 24 h the cell concentration was 1–2 × 10^5^ per well. The cell density should be 80–90% of the well surface, the cells should be evenly distributed over the surface. Before transfection, 5 µL of LipofectamineTM2000 (Invitrogen)/polycation solution was added to 100 µL DMEM without serum and antibiotic. The resulting solution was incubated for 5 min at room temperature. DNA (1–2 µg) was dissolved in 100 µL of DMEM (Dulbecco’s modified Eagles medium) without serum and antibiotic, Lipofectamine TM 2000/polycation solution was added, gently mixed, and incubated at room temperature for 15 min, after which 800 µL of DMEM without serum and antibiotic was added. Before introducing the resulting complex of DNA and LipofectamineTM2000 into the HEK-293 cell culture, the growth medium was removed, the cells were gently washed with 2 mL of DMEM without serum and antibiotic, and the resulting DNA complex with Lipofectamine TM 2000/polycation was added. The transfected cells were incubated at 37 °C in a CO_2_ incubator for 24 h, then 1 mL of DMEM medium containing a twofold concentration of fetal serum and an antibiotic was added.

## 3. Results and Discussion

**Scheme 1:**
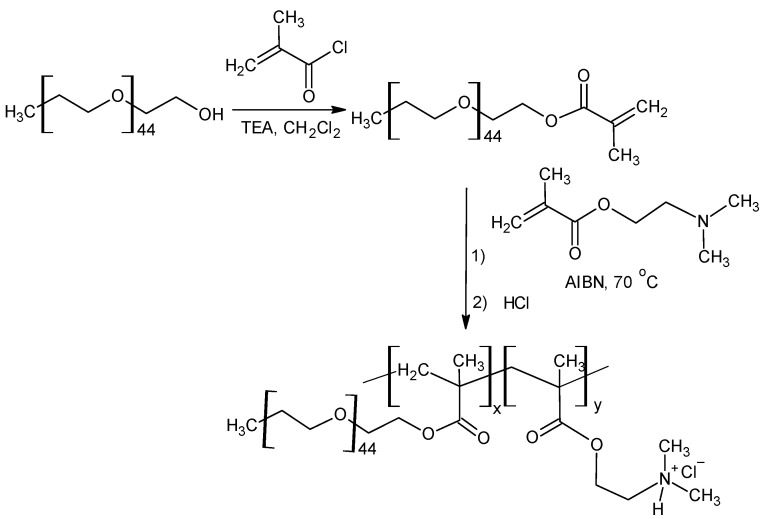
Synthesis of PDMAEMA-co-PEO.

PDMAEMA-co-PEO polyelectrolytes were obtained as a result of radical copolymerization of DMAEMA with polyethylene glycol monomethyl ether methacrylate in various proportions. The last one was synthesized using methacryloyl chloride as acylating agent (Scheme 1):

### 3.1. Study of the Molecular Characteristics of PDMAEMA-co-PEO Polycations by Sedimentation Analysis

The data on molecular weight characteristics, obtained by sedimentation in an ultracentrifuge of PDMAEMA-co-PEO copolymers, are presented in Table 1.

The samples were studied in an aqueous solution of 0.15 M NaCl to suppress the polyelectrolyte effect. Under these conditions, macromolecules of copolymers form compact globules. During the formation of globules, a certain orientation of hydrocarbon chains and polar groups occurs, similar to how it is observed during the formation of micelles from surfactant molecules, i.e., the minimum interfacial tension at the macromolecule–environment boundary is provided [54]. In this case, the more hydrophobic PDMAEMA units are located inside the globules, while the hydrophilic PEO units are located outside. In all samples of copolymers, with an increase in MM, the values of the sedimentation coefficients and the values of hydrodynamic radii of inertia Rg increase, while the values of the diffusion coefficients decrease. From the data of Table 1, one can see a gradual increase in MM in samples 1–3 with an increase in the mole fraction of PDMAEMA units. However, in sample 4, which has the maximum molar part of PEO in this series of samples, we observe the highest molecular mass (MM) value, which may be explained by the formation of micellar associates of PEO macromolecules. It is known from the literature that PEO in water forms associates even at room temperature. Their occurrence can probably be explained by hydrogen bonds and the hydrophobic effect [55].

### 3.2. Study of the Dependence of the Degree of Ionization (α) and Apparent Dissociation Constants pK_app_ of Polycations on the pH of Aqueous Solutions by Potentiometric Titration

**Figure 1:**
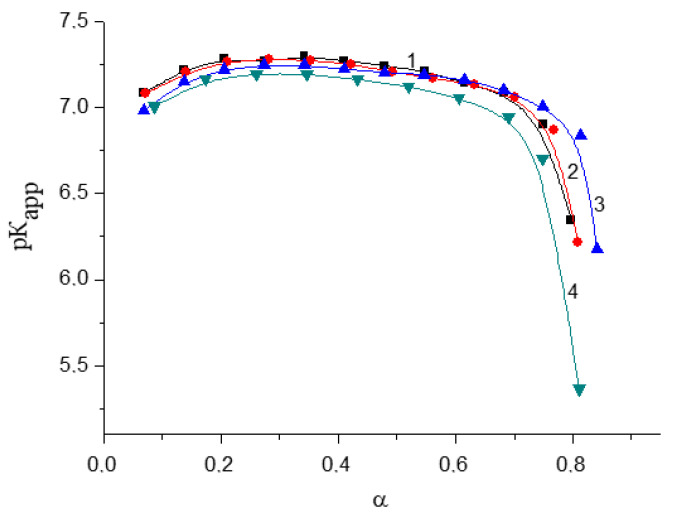
The dependences of the apparent dissociation constants pK_app_ on the degree of ionization (α) of aqueous solutions in 0.15 M NaCl: PDMAEMA-co-PEO-1 (1, black), PDMAEMA-co-PEO-2 (2, red), PDMAEMA-co-PEO-3 (3, blue), PDMAEMA-co-PEO-4 (4, green).

The dependences of the apparent dissociation constants pK_app_ on the degree of ionization (α) of aqueous solutions of PDMAEMA-co-PEO polycations in 0.15 M NaCl are shown in Figure 1.

In Figure 1 the dependences of the apparent dissociation constants pK_app_ of polycations PDMAEMA-co-PEO on the degree of ionization (*α)* of aqueous solutions in 0.15 M NaCl are presented. When comparing the curves, it can be seen that with an increase in the molecular mass of polycations, the pK_app_ values decrease, which may be due to the difference in the molecular mass and conformational structure of these copolymers. It should be noted that all PDMAEMA-co-PEO polycations have similar final values of the degree of ionization (α), except for PDMAEMA-co-PEO-4, which has the highest MM. Moreover, with an increase in the degree of ionization (α), the values of pK_app_ for polycations decrease. This is probably due to the difficulty of the process of attaching each subsequent proton to the amino groups of the polycation with an increase in α, because of the repulsion of like-charged ionogenic groups. 

**Figure 2:**
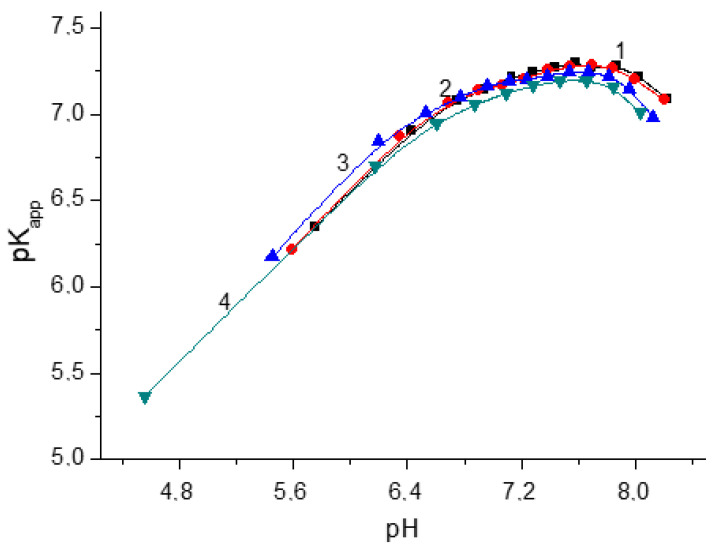
Dependences of the apparent dissociation constant of PDMAEMA-co-PEO on the pH of aqueous solutions in 0.15 M NaCl with a polyelectrolite concentration of 1 mg/mL: PDMAEMA-co-PEO-1 (1, black), PDMAEMA-co-PEO-2 (2, red), PDMAEMA-co-PEO-3 (3, blue), and PDMAEMA-co-PEO-4 (4, green).

The dependences of the apparent dissociation constants pK_app_ of PDMAEMA-co-PEO polycations on the pH of aqueous solutions in 0.15 M NaCl are shown in Figure 2. It should be noted that the final values of pK_app_ of polycations from pH are arranged in order of their MM increasing. If the final pK for polycations PDMAEMA-co-PEO-1-3 are similar in pH: 6.35, 6.22, and 6.18, respectively, then for PDMAEMA-co-PEO-4 pH is 5.3, which is probably due to the formation of associates of macromolecules. Thus, it can be assumed that the process of termination of protonation of ionogenic groups for each of the polyelectrolytes is associated with MM, concentrations of ionogenic groups, and conformational structure. It follows from the above data that an increase in the MM of polycations leads to a longer protonation of the latter, which is an unfavorable factor in the formation of interpolyelectrolyte complexes (IPECs) of DNA with linear polycations.

### 3.3. Study of the Electrophoretic Mobility of Interpolyelectrolyte Complexes of DNA with PDMAEMA-co-PEO Polycations by Laser Microelectrophoresis

**Figure 3:**
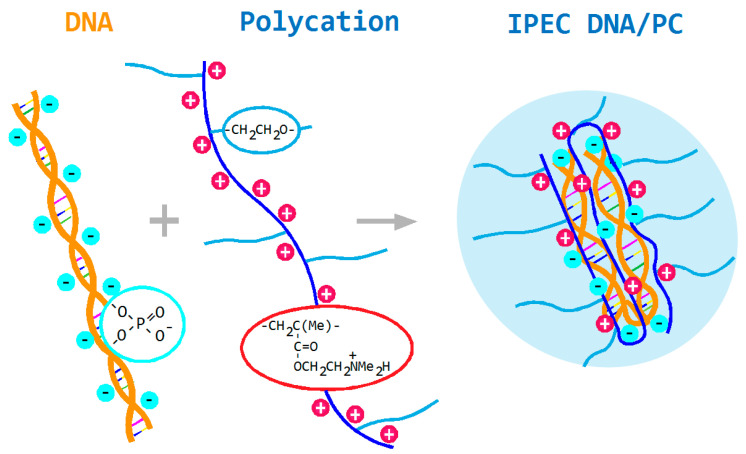
The formation of interpolyelectrolite complexes (IPECs) of DNA with linear polycations.

Interpolyelectrolyte complexes (IPECs) of DNA with linear polycations are formed by mixing solutions of components due to the formation of a cooperative system of interchain electrostatic bonds between the positively charged alkylammonium groups of the polycation chain and the negatively charged phosphate groups of the backbone of DNA. Inclusion in IPEC leads to a significant change in the properties of DNA, including its compaction and stabilization against cleavage by nucleases [40] (Figure 3).

The physicochemical characteristics of IPEC, in particular, solubility, size, and surface charge, strongly depend on its composition, i.e., a base-molar ratio of polycation/DNA in the complex [56]. Inclusion in IPEC leads to a significant change in the properties of DNA, including its compaction and stabilization against cleavage by nucleases. The binding of the polycation to anionic DNA macromolecules leads to the neutralization of the DNA charge, which could be registered by the change in the electrophoretic mobility (EPM) of the resulting IPEC particles. 

**Figure 4:**
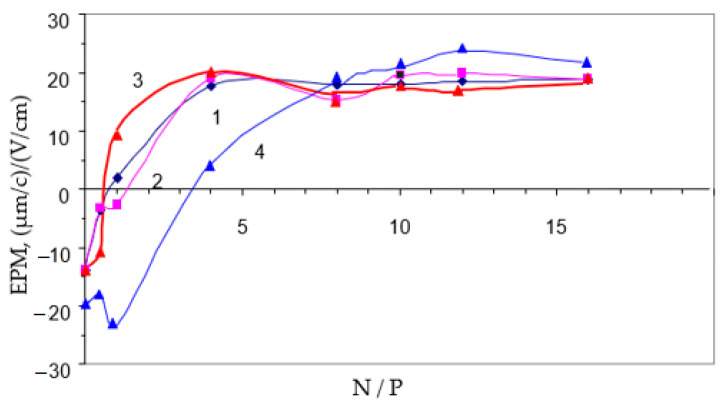
Dependences of EFM of DNA/PC IPEC particles on N/P: PDMAEMA-co-PEO-1 (1, black), PDMAEMA-co-PEO-2 (2, rose), PDMAEMA-co-PEO-3 (3, red), PDMAEMA-co-PEO-4 (4, blue), and [DNA] = 22.5 µg/mL; 10^−2^ M phosphate buffer, pH 7.

Figure 4 shows the results of measuring the EFM of DNA/PC IPEC particles from the ratio γ = [cat]/[an], where [cat] is the concentration of ionogenic groups of the polyelectrolyte and [an] is the concentration of DNA phosphate groups, respectively. The ratio γ can also be denoted as N:P. As expected, in all cases the addition of the polycation solution to the DNA solution was accompanied by the neutralization of its charge (in this case, the EFM of the particles of the complex became equal to zero) and the subsequent recharging of the particles in an excess of the polycation.

It is interesting that the EFM = 0 value for DNA/PC IPEC was achieved at a ratio of N:P = 1. This, in turn, meant almost quantitative binding of the studied polycations to DNA molecules. A further increase in the concentration of the polycation leads to a change in the sign of the IPEC charge, which unambiguously indicates the inclusion of an excess of polycation molecules (N/P > 1) in its composition. The polycation PDMAEMA-co-PEO-4 differs significantly, for which the EFM = 0 value occurred at N/P = 3.3, which can probably be explained by the tendency to form associates of macromolecules of this polycation in solution.

### 3.4. Investigation of the Diameters of Interpolyelectrolyte DNA Complexes with PDMAEMA-co-PEO Polycations by Dynamic Light Scattering

**Figure 5:**
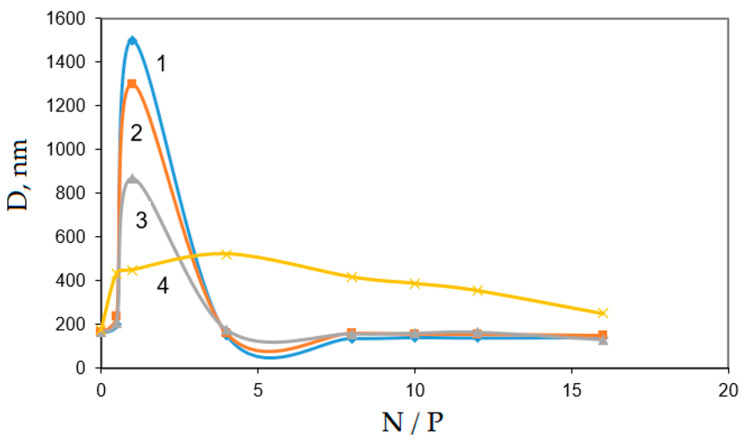
Dependences of the particles diameter of IPEC DNA/PC on N:P: PDMAEMA-co-PEO-1 (1, blue), PDMAEMA-co-PEO-2 (2, orange), PDMAEMA-co-PEO-3 (3, grey), PDMAEMA-co-PEO-4 (4, yellow), and [DNA] = 22.5 µg/mL; 10^−2^ M phosphate buffer, pH 7.

The dependences of the size of DNA/PC IPEC particles on γ for PDMAEMA-co-PEO polycations are shown in Figure 5. It can be seen that with increasing γ, the size of DNA/PC IPEC particles first increases, reaches a maximum at γ = 1 (i.e., when the DNA charge is completely neutralized by the bound polycation), and then decreases to 150–200 nm in excess of the polycation, with the exception of PDMAEMA -co-PEO-4, the size of IPEC with DNA is much larger. Thus, by varying the composition of DNA IPEC with a polycation, it is possible to obtain complex particles of a controlled size that remain stable for a long time. 

### 3.5. Study of the Electrophoretic Mobility of Interpolyelectrolyte Complexes of DNA with the Polycation PDMAEMA-co-PEO-2 by Laser Microelectrophoresis at Different pH Values in Aqueous Solutions

To determine the electrophoretic mobility of interpolyelectrolite complexes with DNA and visualize them by electron microscopy, the PDMAEMA-co-PEO-2 copolymer was chosen, which has an intermediate molecular weight and positive charge density among its other structural analogs, the characteristics of which are given in Table 1.

**Figure 6:**
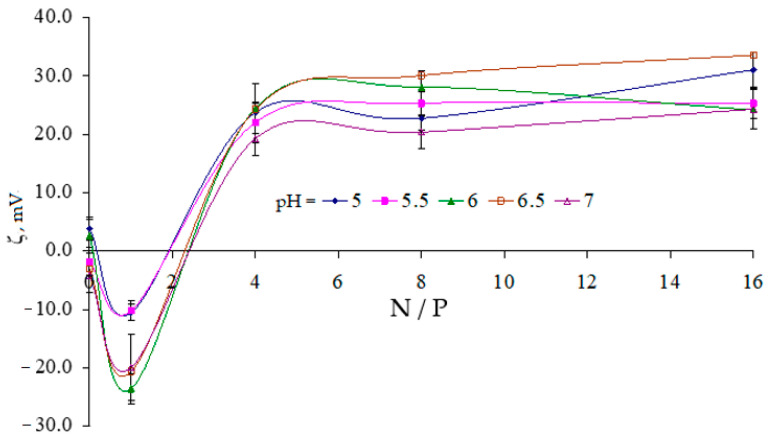
Dependence of the ζ-potential of the IPEC DNA/PDMAEMA-co-PEO-2 complexes on the charge ratio of phosphate groups in the DNA molecule to positively charged units of the polycation (N/P) at different pH values.

The behavior of polycation-DNA complexes in aqueous saline solution was determined by microelectrophoresis (Figure 6). In this case, we were interested in several factors: the change in the charge of the initial DNA in solutions with different pH, and the change in the total charge of the polymer-DNA complexes. The initial charge of DNA fluctuates around the zero value of the surface charge and does not exceed the statistical measurement error. Therefore, we can say that DNA in the considered pH values practically does not change its neutral surface charge. When a polycation is added to DNA when a formal equimolar ratio of nitrogen and phosphate groups (N/P = 1) is reached, unexpected results were obtained: instead of obtaining complexes with a neutral surface charge, due to their stoichiometric ratio, the complexes become negatively charged. Despite the fact that this effect is observed at all pH values, it is nevertheless most pronounced in solutions with pH 6, 6.5, and 7 and reaches a value of −20 mV (ζ-potential). At lower pH = 5 and 5.5 for the same N:P ratio, the ζ-potential value is −10 mV. Further introduction of the polycation leads to a recharging of the surface of the complex (ζ ≈ 20 mV) and, at the ratio N/P = 4, the potential ceases to change (Figure 5). Parallel measurement of the particle diameter shows that at the point N/P = 1, i.e., equivalence of charged groups, the size of the complexes only slightly exceeds the size of the original DNA. A similar picture is also observed with an increase in the ratio of polycation/DNA to 4. A further increase in the ratio of components leads to an increase in the diameter of the complexes. An interesting feature in the behavior of this DNA-polycation system is the fact that the initial introduction of a positively charged copolymer leads to an increase in the negative charge of the surface of the complex as a whole. One possible explanation for this fact may be that in buffers with a given pH, the original DNA remains neutral; phosphate groups are significantly protonated. The addition of a polycation leads to a change in the equilibrium towards the dissociation of DNA units, which leads to the appearance of a negative charge. If this is true, then a decrease in the pH of the solution will lead to an even greater increase in the negative charge due to a decrease in the concentration of protons. This is exactly what we observe from the results of particle charge measurements. On the other hand, at the N/P = 4 ratio the charge becomes strongly positive, but the size of the complexes, as for the stoichiometric ratio, does not significantly exceed the DNA size. A further increase in the introduced polycation (N/P = 8 or 16) leads to an increase in size, but the charge does not change in this case. Two experimental facts: the charge of the complexes at N/P = 8 and 16 has the same sign as the copolymer; the charge of the complexes practically does not change in excess of the polymer. The explanation for the observed facts may lie in the fact that, in addition to the formation of electrostatic DNA-polymer contacts, bonds are formed (hydrogen, hydrophobic, etc.) that additionally stabilize the polycation-DNA complex.

**Figure 7:**
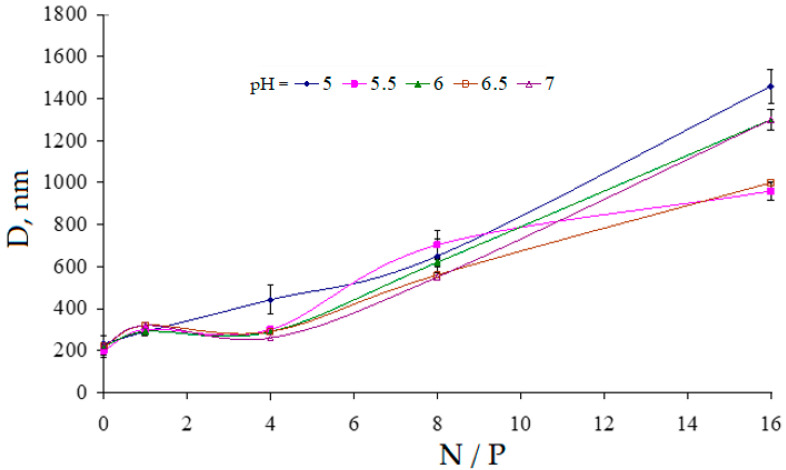
Dependence of the diameter of DNA IPEC/PDMAEMA-co-PEO-2 complexes on the charge ratio of phosphate groups in the DNA molecule to positively charged units of the polycation (N/P) at different pH values.

Interestingly, at pH 5.5 in aqueous solutions, the diameters of DNA/PC IPECs increased (Figure 7). This probably occurs as a result of further protonation of the amino groups of the polycation, which leads to an increase in the size of the polyelectrolyte macromolecules. The point corresponding to the final value of pK_app_ of the polycation means the end of the process of proton addition to the polycation. It should be noted that each specific pH value of an aqueous solution corresponds to a specific conformation (space arrangement of macromolecular units) of the polycation. During transfection, DNA/PC complex particles enter the cell with a more acidic environment. As mentioned above, when studying the change in DNA/PC IPEC diameters with different pH values, this leads to a change in the conformation of polyelectrolyte macromolecules. It can be assumed that as a result of the cooperative effect, the process of DNA release from PK macromolecules becomes possible.

### 3.6. Study of the Morphology of Interpolyelectrolyte Complexes of DNA with Polycation PDMAEMA-co-PEO-2 by Transmission Electron Microscopy

**Figure 8:**
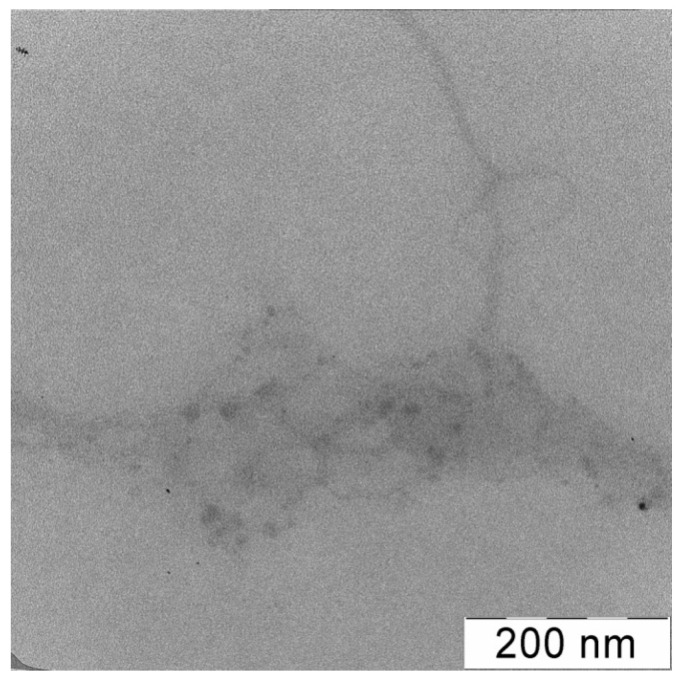
Electron micrograph of IPEC DNA/PDMAEMA-co-PEO-2, 10^−2^ M phosphate buffer, pH 7, ratio N/P = 4:1.

**Figure 9:**
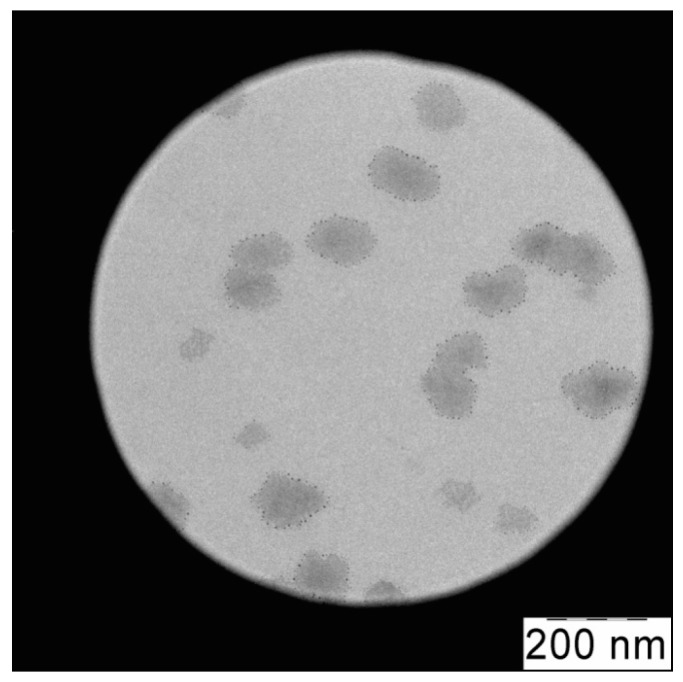
Electron micrograph of IPEC DNA/PDMAEMA-co-PEO-2, 10^−2^ M phosphate buffer, pH 7, ratio N/P = 8:1.

Electron micrographs of IPEC DNA/PDMAEMA-co-PEO-2 with different N:P contents are shown in Figure 8 and Figure 9. At a ratio of N/P = 4:1, the IPEC micrograph (Figure 8) shows both circular DNA fragments and filamentous ones covered with polycation macromolecules. 

With an increase in the polycation ratio in IPEC DNA/PDMAEMA-co-PEO-2 to N/P = 8:1 (Figure 9), irregularly shaped structures with a dark core inside are formed. Along with individual DNA/PC IPEC particles, the photomicrograph shows doubled complex particles with a common surface boundary. The size of single particles of IPEC DNA/PC is from 50 to 150 nm, and of doubled particles up to 200 nm.

### 3.7. Study of the Process of Transfection of Interpolyelectrolyte Complexes with Polycations PDMAEMA-co-PEO

**Figure 10:**
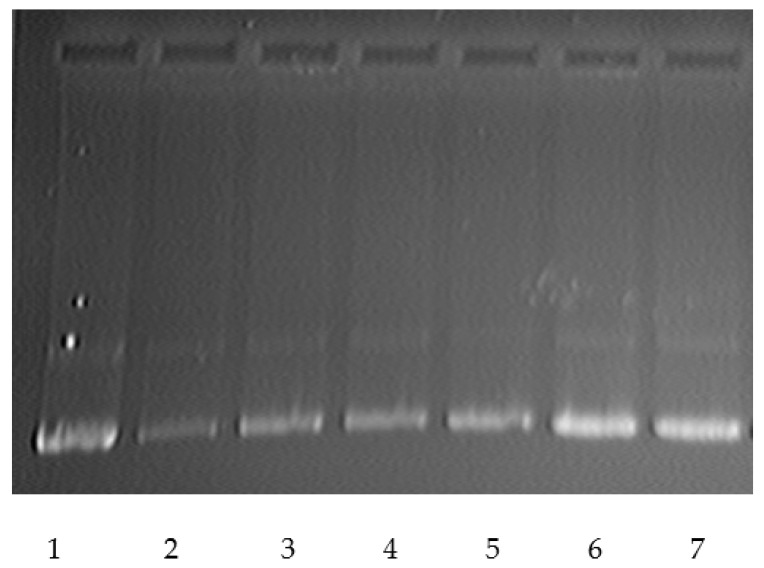
Electrophoresis in 1% agarose gel of free DNA (control) and IPEC DNA/PDMAEMA-co-PEO-2 after treatment with the DNA complex. Control: plasmid DNA pVAX1/LacZ (0.1 µg) (1); (pVAX1/LacZ+pDMAE-co-PEO-2) = IPEC; IPEC at: N/P = 1:1 (2); N/P = 2:1 (3); N/P = 4:1 (4); N/P = 6:1 (5); N/P = 8:1 (6); and N/P = 10:1 (7).

The inclusion of DNA in the interpolyelectrolyte complex leads to a significant change in its properties, including its compaction and stabilization against cleavage by nucleases. The resistance of the IPEC DNA/PC complex to nucleases was assessed by agarose gel electrophoresis (1%). After electrophoresis, the gel was stained with ethidium bromide solution (Figure 10). In ultraviolet light, DNA-EtBr emits orange light.

**Figure 11:**
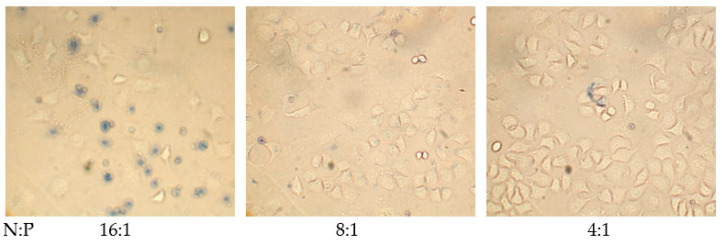
Cytotoxicity of PDMAE-co-PEO-2 at various ratios of N:P.

**Figure 12:**
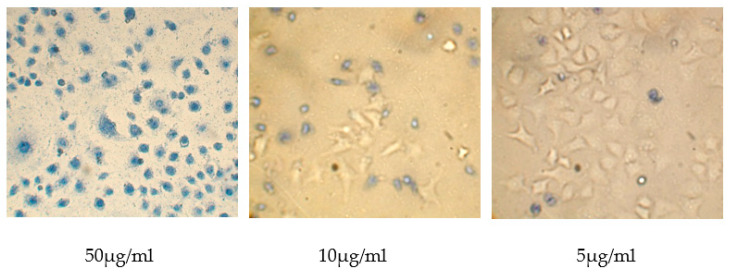
Cytotoxicity of PDMAEMA-co-PEO-2 at various dilutions (trypan blue stain).

The data obtained make it possible to choose the ratio of the complex components (N/P above 6:1) when the DNA macromolecules are completely covered with polycations. This is important because, firstly, it allows the protection of DNA from degradation by nucleases. Secondly, the size of complexes less than 200 nm should not affect transfection into the cell. The positive surface charge of the complexes at a ratio of components greater than (N/P = 6:1) will facilitate their interaction with cells that hold a negative charge. When studying the cytotoxic effect of polymers in vitro, a certain stimulating effect on the L929 cell line of polycations was observed at low concentrations (1 μg/mL, 5 μg/mL); an increase in the concentration of cationic polymers causes damage to the cell membrane and leads to a decrease in the number of surviving cells Figure 11. The toxic effect of polymer/DNA complexes is determined by their structure, hydrophobicity, type of cationic groups, charge density, and size. An increase in the charge of the polymer/DNA complex (an increase in the N/P ratio of more than 10:1) in some cases leads to a noticeable decrease in surviving cells (Figure 12).

**Figure 13:**
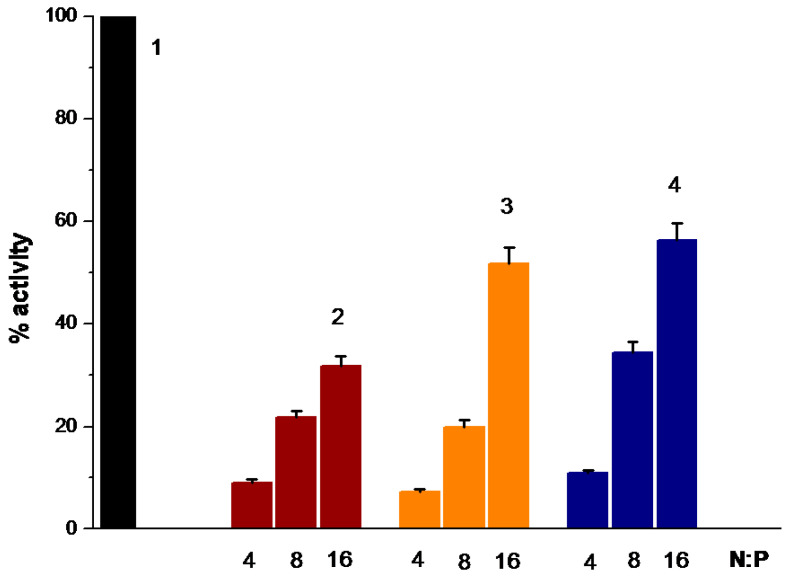
IPEC DNA/PC transfection results: Lipofectamine (1); PDMAEMA-co-PEO-1 (2); PDMAEMA-co-PEO-2 (3); and PDMAEMA-co-PEO-4 (4) at different N/P ratios.

The penetration (transfection) efficiency of DNA IPEC with PDMAEMA-co-PEO polycations was studied in eukaryotic HEK 293 cells in vitro. The results of transfection with IPEC DNA/PC are shown in Figure 13 in comparison with lipofectamine—a commercial drug. The best transfection results among DNA/PC IPECs are for PDMAEMA-co-PEO-1 and PDMAEMA-co-PEO-2, which have lower molecular weights and, as a result, smaller sizes of DNA/PC IPECs. Completion of the process of protonation of polycations at a pH value of 5–6 is a favorable factor in the release of DNA in eukaryotic cells. In terms of transfection efficiency, DNA IPECs with pDMAEMA-co-PEO polycations are inferior to the commercial drug, lipofectamine, but are less toxic than lipofectamine. It should be noted that DNA IPECs with polycations are stable for a longer time, in contrast to DNA complexes with lipofectamine.

## 4. Conclusions

Interpolyelectrolyte complexes based on pDMAEMA-co-PEO polycations and circular DNA have been obtained. The transfection efficiency of DNA IPEC with PDMAEMA-co-PEO polycations was studied in eukaryotic HEK 293 cells in vitro. 

The best transfection results among DNA/PC IPECs are for PDMAEMA-co-PEO-1 and PDMAEMA-co-PEO-2, which have lower molecular weights and, as a result, smaller sizes of DNA/PC IPECs. In terms of transfection efficiency, DNA IPECs with pDMAEMA-co-PEO polycations are inferior to the commercial drug, lipofectamine, but are less toxic than lipofectamine. In addition, DNA IPECs with polycations are stable for a longer time, in contrast to DNA complexes with lipofectamine. 

The toxic effect of polymer/DNA complexes is determined by their structure, hydrophobicity, type of cationic groups, charge density, and size. A decrease in the charge of the polymer/DNA complex (a decrease in the N/P ratio below 10:1) leads to a noticeable increase in cell survival.

The results of the study of the obtained DNA/PC IPECs suggest that the polycations PDMAEMA-co-PEO-1 and PDMAEMA-co-PEO-2 will be of particular interest as a means of gene delivery in experiments in vivo. 

## Figures and Tables

**Table 1:** Molecular weight characteristics of copolymers and sedimentation data.

Polymer	y/x *	S_0_ × 10^13^, c	D_0_ × 10^7^, cm^2^/c	υ, cm^3^/g	M_SD,_ kD	R_g_, nm	C mmol/g
PDMAEMA-co-PEO-1	78	2.35	2.43	0.847	165	10	5.47
PDMAEMA-co-PEO-2	170	2.7	2.1	0.860	228	12	5.9
PDMAEMA-co-PEO-3	330	2.97	1.5	0.840	392	17	6.11
PDMAEMA-co-PEO-4	40	5.26	1.5	0.845	620	23	4.8

* determined by using the ratio of NMR ^1^H signals integral intensities of δ = 4.01 ppm (OCH_2_) and δ = 3.61 ppm (CH_2_CH_2_O).

## Data Availability

The characterization data are available upon request from the authors.
